# An examination of three prescribing cascades in a cohort of older adults with dementia

**DOI:** 10.1186/s12877-021-02246-2

**Published:** 2021-05-08

**Authors:** Shanna C. Trenaman, Susan K. Bowles, Susan Kirkland, Melissa K. Andrew

**Affiliations:** 1 Department of Medicine (Geriatrics), Dalhousie University, Halifax, Nova Scotia Canada; 2 Nova Scotia Health, Halifax, Nova Scotia Canada; 3 College of Pharmacy, Dalhousie University, Halifax, Nova Scotia Canada; 4 Department of Community Health and Epidemiology, Dalhousie University, Halifax, Nova Scotia Canada

**Keywords:** Dementia, Prescribing cascades, Polypharmacy, Geriatrics

## Abstract

**Background:**

Prescribing cascades are a source of inappropriate prescribing for older adults with dementia. We aimed to study three prescribing cascades in older Nova Scotians with dementia using administrative databases.

**Methods:**

Cohort entry for Nova Scotia Seniors’ Pharmacare Program beneficiaries was the date of dementia diagnosis. Prescription drug dispensing data was extracted for inciting medication and second treatment (cholinesterase inhibitor and bladder anticholinergic, metoclopramide and Parkinson’s disease medication, or calcium channel blocker (CCB) and diuretic) over the six-year period April 1, 2009 to March 31, 2015. In three separate analyses, dispensing an inciting medication signaled a look back of 365 days from the date of first dispensing to confirm that the second treatment was started after the inciting medication. The prescribing cascade was considered when the second treatment was started within 180 days of the inciting treatment. Sex differences in the prescribing cascade were tested using t-tests or chi square tests as appropriate. Both univariate (unadjusted) and multivariate (adjusted) logistic regression (adjusted for age, rurality, and sex) and Cox proportional hazards regression was used to identify risk factors for the prescribing cascade.

**Results:**

From March 1, 2005 to March 31, 2015, 28,953 Nova Scotia Seniors' Pharmacare beneficiaries with dementia (NSSPBD) were identified. There were 60 cases of bladder anticholinergics following cholinesterase inhibitors, 11 cases of Parkinson’s disease medication following metoclopramide, and 289 cases of a diuretic following CCB in the cohort. Regression analysis demonstrated that risk of bladder anticholinergics following cholinesterase inhibitors and diuretics following CCBs were associated with female sex. Cox regression suggested that bladder anticholinergics were less often used by those on cholinesterase inhibitors and did not identify CCB use as leading more frequently to diuretic use.

**Conclusions:**

The combination of diuretics following CCB was the most common prescribing cascade and bladder anticholinergics following cholinesterase inhibitors the second most common. However, exposure to the inciting medications did not increase risk of exposure to the second treatments. Combinations of bladder anticholinergics following cholinesterase inhibitors and diuretics following CCBs were more common for women raising concern that women may be at increased risk of these prescribing cascades.

## Background

The concept of the prescribing cascade was first reported by Rochon and Gurwitz in 1995 [1]. It is defined as an adverse drug event misinterpreted as a new medical condition resulting in a new medication being prescribed to treat the adverse drug event [1–3]. Prescribing cascades can affect people of any age [4–7] but most commonly occur in older adults [6, 8, 9]. This is related to the high level of medication use in older adults [10, 11]. Polypharmacy [12] is associated with increased incidence of adverse drug events [13, 14] which may provide opportunities for prescribing cascades to occur. Older adults with dementia are more susceptible to adverse drug events than similarly aged controls without dementia due to their higher level of medication use [15–19].

Cholinesterase inhibitors, used to manage symptoms of dementia, exert their therapeutic activity by binding and inhibiting the cholinesterase enzyme [20, 21], increasing the concentration of available acetylcholine [22–24]. As cholinesterase inhibitors are not selective for the brain, acetylcholine concentrations are increased systemically. Activation of muscarinic receptors [25, 26] in the urinary tract causes forceful contraction of the detrusor muscle which may result in urge type urinary incontinence. Treating this iatrogenic urinary incontinence with a bladder anticholinergic is thus a prescribing cascade.

Metoclopramide is used to treat nausea, vomiting, diabetic gastroparesis, or loss of appetite; all of which can occur in older adults. Metoclopramide’s mechanism of action is to antagonize the activity of dopamine at receptors in the chemoreceptor trigger zone, located outside the blood-brain barrier, and in the gut [27]. Metoclopramide is non-selective and notably, crosses the blood brain barrier allowing for inhibition of dopamine receptors within the brain. This inhibition can induce Parkinsonian type movement changes. These symptoms can be mistaken for development of Parkinson’s Disease or vascular pseudoparkinsonism and if this leads to treatment with dopaminergic medications it represents a prescribing cascade.

Calcium channel blockers (CCB) are recommended as initial monotherapy or combination therapy for adults with either diastolic or systolic hypertension [28, 29]. CCBs are associated with the adverse event of pedal edema (prevalence 2–25%) which is due to vasodilation of peripheral arterioles and depends on drug, dose, duration and sex [30–33]. As CCB-induced edema is dose dependent [30, 34, 35], and older adults who experience alteration in drug pharmacokinetics may experience increased CCB drug exposure, they may also be at a greater risk of this adverse event [36]. Treating primary care providers may mistake CCB-induced pedal edema for symptoms of heart failure or peripheral vascular disease and initiate a prescribing cascade by prescribing diuretics to treat the CCB-induced edema [6, 37].

Identifying the presence of prescribing cascades is an important component of addressing potentially inappropriate medication use by older adults which helps avoid both increased cost of medications, and sequelae of avoidable adverse drug events [2, 3, 38]. We aimed to determine the incidence of and risk factors for these three prescribing cascades in a population of older adults with dementia in Nova Scotia, Canada.

## Methods

### Data sources

Administrative claims data were made available through Health Data Nova Scotia (HDNS) following REB approval (File #: 1023625). Data were extracted from Medical Services Insurance (MSI) Physician’s Billings (MED), Seniors Pharmacare (PHARM), Vital Statistics (VITAL), and the Canadian Institute for Health Information – Discharge Abstract Database (DAD). The data set included administrative data from March 1, 2005 to March 31, 2018. Databases used were housed by HDNS and data linkage was done by a third party using MSI health card number, which was not available to the research team but instead was replaced with a study identification number.

### Cohort definition

The cohort was drawn from beneficiaries of the Seniors' Pharmacare program. This provincial medication insurance program is available to all Nova Scotian older adults as of their 65th birthday and participation is voluntary. Nova Scotia Seniors' Pharmacare is available to community-dwelling adults, those residing in a long-term care facility, or those in alternate living arrangements such as group homes, etc. Prescription data is from those prescrptions filled in a community pharmacy. Prescriptions filled in hospitals are not captured. Any adults over 65 years of age with who did not participate in the Nova Scotia Seniors' Pharmacare Program (approximately 63% of adults over 65 years of age participate in the Nova Scotia Seniors' Pharmacare Program [39]) were not captured in the analysis. Reasons for non-subscription to Seniors' Pharmacare would include existing private drug insurance coverage (e.g., from previous employment and pension plans) or not feeling that drug coverage was needed. Cohort entry was from the date that a Seniors' Pharmacare beneficiary had at least one occurrence of any one of the International Classification of Diseases Clinical Modification (ICD) 9/10 codes that identify dementia from the MED or DAD databases in the entry event window between March 1, 2005 to March 31, 2015 (Table 1). The ICD codes chosen were identified in the Nova Scotia Dementia Strategy [40] as the most complete method to identify cases of dementia using locally available administrative data. Cohort exit was at the date of death or March 31, 2015. The MED database provided date of dementia diagnosis, and age at first appearance of a dementia diagnosis, and the VITAL database provided the date of death. The PHARM database provided prescription drug dispensing data for requested drug classes (Table 2) over the six-year period from April 1, 2009 to March 31, 2015 including medication (ATC code), quantity dispensed, prescription fill date, sex, and geographic location specified as urban or rural based on the second digit of the postal code [41]. We did not capture data regarding medication dose or prescriber.

**Table 1:** ICD 9/10 diagnosis codes that were identified by the Nova Scotia Dementia Strategy as most likely to identify an individual with a diagnosis of dementia using administrative claims data

Description	ICD-9	ICD-10
Alcohol-induced persisting amnestic disorder	290.x	F01.x, F05.1
Alcohol-induced persisting dementia	291.1	F10.6
Amnestic disorder in conditions classified elsewhere	291.2	F10.7
Dementia in conditions classified elsewhere	294.0	F04.x
Other cerebral degenerations ***Includes****: Alzheimer’s disease; Frontotemporal dementia; Senile degeneration of the brain; Communicating hydrocephalus; Idiopathic normal pressure hydrocephalus; Cerebral degeneration in diseases classified elsewhere; dementia with Lewy’s bodies; Dementia with Parkinsonism; Cerebral degeneration, unspecified.* ***Excludes****: Obstructive hydrocephalus; Reye’s syndrome*	331.0–331.3, 331.5–331.7, 331.82, 331.83, 331.89, 331.9	G30.x, G31.0, G31.1, G31.8, G31.9, G32.8, G91.0, G91.2-G91.3, G91.8, G91.9, G94.x
Senility without mention of psychosis	797	R54.x

**Table 2:** Prescribing Cascade Medications

Prescribing Cascade	Inciting Medication	Second Treatment
1	donepezil (N06DA02) rivastigmine (N06DA03) galantamine (N06DA04)	oxybutynin (G04BD04) tolterodine (G04BD07) solifenacin (G04BD08) trospium (G04BD09) darifenacin (G04BD10) fesoterodine (G04BD11)
2	metoclopramide (A03FA01)	levodopa-carbidopa (N04BA02) levodopa-carbidopa-entacapone (N04BA03) amantadine (N04BB01) ropinirole (N04BC04) pramipexole (N04BC05) selegiline (N04BD01) entacapone (N04BX02)
3	amlodipine (C08CA01) nifedipine (C08CA05) felodipine (C08CA02	furosemide (C03CA01) spironolactone (C03DA01) hydrochlorothiazide (C03EA01) amiloride (C03DB01) ethacrynic acid (C03CC01) bumetanide (C03CA02)

### Analytic procedures

Individuals from the cohort were followed from April 1, 2010 or cohort entry until they had a dispensing of any one of the three inciting medications (Table 2). Once an inciting medication was dispensed (cholinesterase inhibitor, metoclopramide or CCB) there was a look back of 365 days from the first dispensing date to confirm that the second treatment (bladder anticholinergic, Parkinson’s disease medication or diuretic) was not started until after the inciting medication. The look back period of 1 year was chosen as it was unlikely treatment for an existing condition would remain untreated for more than a year under routine circumstances. A 1 year look back period has been used in previous analyses of prescribing cascades [42] but there have been look-back periods as short as 6 months in employ [9]. Once confirmed that the second treatment was not used in the year preceding the inciting medication the individual was followed forward from the first dispensing of the inciting medication to identify an overlapping prescription with the second treatment. If the second treatment was started up to 180 days (6 months) after the first dispensing of the inciting medication this was identified as an instance of the prescribing cascade. If a medication was started more than 6 months after the inciting medication it was considered unlikely to be related to the inciting medication and more likely related to a new indication or medical condition. However, some previous research has allowed an interval of 1 year between inciting medication and second treatment and still considered the event an occurrence of the prescribing cascade [42–44]. The 180 day timeframe chosen was consistent with the amount of time required for any of the three inciting medications to cause their associated adverse event; urinary incontinence due to cholinesterase inhibitors typically presents within 26 weeks (182 days) of medication initiation [45, 46], metoclopramide carries a black box warning that more than 12 weeks of use may cause tardive dyskinesia [47, 48] and although metoclopramide induced movement disorders have been considered to occur as much as 4 years after drug initiation [49] we chose to be consistent with a 180 day interval between treatments; and CCB induced pedal edema occurs typically within the first 12 weeks of treatment [50, 51]. Robustness of the prescribing cascade definition was tested by extending the window from 180 days to 365 days and reducing the window to 90, 60 and 30 days. We explored other periods between medications to assess the lag between inciting medication and second treatment. Sex differences in the prescribing cascade were tested using t-tests or chi square tests as appropriate. Both univariate (unadjusted) and multivariate (adjusted) logistic regression (adjusted for age, rurality, and sex) was used to identify risk factors for the prescribing cascade.

In a secondary analysis we considered being dispensed the prescribing cascade implicated drug (second treatment) as an outcome of interest in a survival analysis using Cox proportional hazard models to examine hazard ratios. This method allows comparison of those who receive inciting medication with those who do not and the likelihood of initiating the second treatment. Time to event was considered from date of the inciting medication prescription to the dispensing of the prescribing cascade-implicated drug, with comparisons made for those dispensed and not dispensed an inciting medication within the time frame. Missing data were handled using case-wise deletion.

### Ethics approval

This retrospective study received ethics approval from the Nova Scotia Health Research Ethics Board (REB File #: 1023625), with subsequent renewals. The research study was approved by the Health Data Nova Scotia Data Access Committee.

### Statistical software

All data analysis was completed on STATA version 15.1, StataCorp, Lakeway Drive, College Station, Texas, USA.

## Results

In the period from April 1, 2010 to March 31, 2015, a total of 28,953 Nova Scotia Seniors' Pharmacare Beneficiaries with dementia (NSSPBD) (62% women) were identified as receiving a dementia diagnosis. The average age at dementia diagnosis was 81.1 years (95% confidence interval (CI): [81.0–81.2]) with the mean age of women being 2.5 years (95% CI: [2.9–3.6]) older than men at dementia diagnosis (*p* < 0.0001). Details on cohort sex, age and geographic location are provided in Table 3.

**Table 3:** Details of the total cohort of Nova Scotia Seniors’ Pharmacare Beneficiaries with Dementia (NSSPBD)

	Total	Women	Men
Number NSSPBD	28,953	17,946 (62.0%)	10,529 (36.4%)
Mean age at diagnosis in years (95% CI)	81.1 (81.0–81.2)	82.1 (82.0–82.2)	79.6 (79.4–79.7)
Age 65.0–74.9 (% cohort)	6355 (21.9%)	3266 (11.2%)	2922 (10.0%)
Age 75.0–84.9 (% cohort)	12,222 (42.2%)	7412 (25.6%)	4637 (16.0%)
Age 85.0–94.9 (% cohort)	8254 (28.5%)	5764 (19.9%)	2385 (8.2%)
Age 95+ (% cohort)	808 (2.8%)	637 (2.2%)	159 (0.5%)
Urban dwelling (%)	18,485 (63.8%)	11,812 (40.8%)	6673 (23.0%)
Rural dwelling (%)	9342 (32.3%)	5806 (20.1%)	3536 (12.2%)

### Cascade 1: Cholinesterase Inhibitor & Bladder Anticholinergic

A total of 117,416 cholinesterase inhibitor (ATC N06DA) prescriptions were dispensed to 5772 NSSPBD (68.7% women and 19.9% of NSSPBD) over the period of investigation. Cholinesterase inhibitors used were donepezil (57.0%), galantamine (36.0%), and rivastigmine (7.0%). NSSPBD receiving cholinesterase inhibitor treatment used these agents on average 1.9 (95% CI [1.8–1.9]) years with women using cholinesterase inhibitors for longer durations (1.9 vs. 1.8 years, *p* = 0.0012) (Table 4).

**Table 4:** Detailed drug utilization: Details of cohort for those who experienced a prescribing cascade

Prescribing Cascade	1		2		3	
Medication class	Cholinesterase inhibitor (N06DA)	Bladder Anticholinergic (G04BD)	Metoclopramide (*A03FA01*)	Parkinson’s Disease medication (*N04B*)	CCB (*C08CA*)	Diuretic (*C03*)
Number of cases of prescribing cascade	60		11		289	
Mean age at dementia diagnosis for NSSPBD in prescribing cascade, years (SD)	79.0 (7.4)		80.9 (8.0)		82.8 (7.7)	
Implicated medication	Donepezil – 40 (66.7%) Galantamine, Rivastigmine – 20 (33.3%)	Oxybutynin – 43 (71.7%) Tolterodine, Solifenacin, Trospium, Darifenacin, Fesoterodine – 28.3%	Metoclopramide – 11 (100%)	Levodopa and decarboxylase inhibitor, Ropinirole, Pramipexole – 11 (100%)	Amlodipine – 193 (66.8%) Felodipine – 10 (3.5%) Nifedipine – 86 (29.8%)	Furosemide – 224 (77.5%) Hydrochlorothiazide – 41 (14.2%) Ethacrynic acid, Spironolactone, Amiloride – 24 (8.3%)

*NSSPBD* Nova Scotia Seniors’ Pharmacare Beneficiaries with Dementia, *SD* standard deviation

There were 17,806 prescriptions for bladder anticholinergics (ATC G04BD) dispensed to 1263 NSSPBD (73.0% women and 4.4% of NSSPBD). Bladder anticholinergics used were oxybutynin (73.2%), tolterodine (14.5%), solifenacin (8.2%), trospium, darifenacin, and fesoterodine (combined 4.1%). For all bladder anticholinergic users in the cohort duration of bladder anticholinergic use was similar for men and women (1.3 years, *p* = 0.4).

The accepted definition of the prescribing cascade, where cholinesterase inhibitor preceded the bladder anticholinergic by up to 6 months (180 days), was identified in 60 cases (41 women and 19 men, 0.2% of NSSPBD) (Table 4). Shortening the window for the prescribing cascade to: 90 days reduced the number of identified cases to 36 (25 women, 11 men, 0.1%), 60 days reduced the number of identified cases to 32 (21 women, 11 men, 0.1%) and 30 days reduced the number of identified cases to 16 (10 women, 6 men). Extending the window to 365 days resulted in 52 additional cases (112 cases, 80 women, 32 men, 0.4%).

Univariate and multivariate cross-sectional analysis with logistic regression (Table 5) suggested that age and sex were statistically significant risk factors for occurrence of the prescribing cascade with younger age and female sex being associated with the increased use of bladder anticholinergics following cholinesterase inhibitors. In unadjusted and multivariate Cox regression, those dispensed cholinesterase inhibitors were found to have a lower risk of subsequently receiving a bladder anticholinergic medication (unadjusted hazard ratio [HR], 0.77; 95% CI, 0.68–0.87; adjusted HR, 0.79; 95% CI, 0.68–0.92) (Table 6).

**Table 5:** Cross-sectional analysis Logistic Regression (unadjusted and adjusted) for risk factors for prescribing cascades

	Covariates	Unadjusted Odds Ratio (95% CI)	Adjusted Odds Ratio (95% CI)
Bladder anticholinergics following cholinesterase inhibitors	Age at diagnosis (years)	**0.94 (0.91–0.97)**	**0.93 (0.90–0.97)**
	Rurality (Urban)	0.88 (0.51–1.5)	0.92 (0.53–1.58)
	Sex (Male)	**0.54 (0.30–0.99)**	**0.46 (0.25–0.85)**
Diuretic following CCB	Age at diagnosis (years)	**1.01 (1.00–1.02)**	1.01 (0.99–1.03)
	Rurality (Urban)	1.17 (0.91–1.5)	1.14 (0.88–1.48)
	Sex (Male)	**0.67 (0.51–0.88)**	**0.0033 (0.00090–0.012)**

**Table 6:** Hazard Event Rates Ratios for prescribing cascades

Main Analysis (Full Cohorts)	Cholinesterase Inhibitor User (***n*** = 5772)	No Cholinesterase Inhibitor (***n*** = 13,770)
Total Number (%) of events (newly dispensed bladder anticholinergic)	366 (6.3%)	868 (6.3%)
Duration of Follow-up, mean ± SD, days	525 ± 75.3	332 ± 27.7
Unadjusted HR (95% CI)	0.77 (0.68–0.87)	1.00 (Referent)
Adjusted HR (95% CI) (age, sex, rural/urban)	0.79 (0.68–0.92)	1.00 (Referent)
**Main Analysis (Full Cohorts)**	**CCB User (*n*** **= 4639)**	**No CCB (*n*** **= 14,903)**
Total Number (%) of events (newly dispensed diuretic)	1708 (36.8%)	4536 (30.4%)
Duration of Follow-up, mean ± SD, days	653.1 ± 24.6	651.3 ± 17.4
Unadjusted HR (95% CI)	1.00 (0.94–1.05)	1.00 (Referent)
Adjusted HR (95% CI) (age, sex, rural/urban)	1.04 (0.98–1.10)	1.00 (Referent)

*CI* confidence interval, HR hazard ratio, *SD* standard deviation

### Cascade 2: Metoclopramide & Antiparkinsonian Agents

In total 3760 prescriptions for metoclopramide (ATC A03FA01) were dispensed to 1038 NSSPBD (76.7% women and 3.6% of NSSPBD). NSSPBD who received at least one dispensing of metoclopramide were on average 81.6 years of age at the time of their dementia diagnosis and 84.9 years of age at the time of their first metoclopramide prescription. Those NSSPBD receiving metoclopramide used it for an average 0.2 years or 2.4 months. Duration of use did not differ between men and women (0.2 versus 0.2 years, *p* = 0.29) (Table 4).

25,984 prescriptions for Parkinson’s Disease medications (ATC N03) were dispensed to 997 NSSPBD (53.8% women and 3.4% of NSSPBD). NSSPBD who received at least one prescription for a Parkinson’s Disease medication were on average 78.6 years of age at the time of their dementia diagnosis and 81.0 years at the time of their first Parkinson’s Disease medication prescription. Parkinson’s Disease medications used included levodopa and decarboxylase inhibitor (74%), pramipexole (12.2%), ropinirole (3.8%), entacapone (3.3%), amantadine (2.8%), selegiline (2.0%), and levodopa, decarboxylase inhibitor and COMT inhibitor (1.1%). Those NSSPBD receiving Parkinson’s Disease treatment used it for on average 2.3 years (95% CI: [2.1–2.5]) with no statistically significant difference in duration between men and women (2.4 versus 2.2, *p* = 0.059) (Table 4).

The accepted definition of the prescribing cascade, where metoclopramide preceded the Parkinson’s Disease medication by less than 6 months (180 days), was identified in only 11 cases (Table 4). Due to the very low number of cases of the prescribing cascade it was not possible to perform a logistic or cox regression analysis to identify risk factors or a sex-difference for this prescribing cascade. Extending to a year identified an additional 5 possible cases (total 16, 0.06% NSSBPD).

### Cascade 3: Calcium Chanel Blocker & Diuretic

In total 93,688 prescriptions for a CCB (ATC C08CA) were dispensed to 4639 NSSPBD (71.4% women and 16.0% of NSSPBD). NSSPBD who received at least one dispensing of a CCB were on average 81.3 years of age at the time of their dementia diagnosis. Women who received at least one prescription for a CCB were on average 4.8 years older than men at their dementia diagnosis (82.6 versus 77.8 years, *p* < 0.0001). The average age NSSPBD initiated CCB at 83.5 years but women were also older than men at the time of their first CCB prescription by a mean 5.1 years (85.0 versus 79.9, *p* < 0.0001)). CCB prescriptions were most commonly for amlodipine (67.4%), followed by felodipine (3.9%), and nifedipine (31.3%) (Table 4).

There were 117,692 prescriptions for diuretic medications dispensed to 6389 NSSPBD (70.6% women and 22.1% of NSSPBD). NSSPBD who received at least one prescription for a diuretic were on average 82.4 years of age at the time of their dementia diagnosis. At their first diuretic prescription NSSPBD were on average 84.8 years and women were older than men (86.1 versus 81.8 years (p < 0.0001)). Diuretics used by NSSPBD included furosemide (86.0%), spironolactone (5.2%), hydrochlorothiazide (6.5%), amiloride, ethacrynic acid and bumetanide (0.4%) (Table 4).

The accepted definition of the prescribing cascade, where CCB prescription preceded the diuretic medication prescription by less than 6 months (180 days), was identified in 289 cases. This represents 1.0% of all NSSPBD and 6.0% of NSSPBD who used CCB (Table 4). Shortening the window for the prescribing cascade to 90, 60 or 30 days reduced the number of cases of the prescribing cascade to 238, 202, and 130 respectively. Extending the window to 365 days resulted in 369 cases (250 women, 96 men, 23 with sex missing, 1.3% NSSPBD).

Logistic regression (Table 5) showed that in unadjusted analysis those of older age and women were at an increased risk of the prescribing cascade. In adjusted analysis (female) sex was the only covariate that was statistically significantly associated with increased for risk of the prescribing cascade. In unadjusted and multivariate Cox regression, those dispensed CCB were not found to have a different risk of subsequently receiving a diuretic (unadjusted hazard ratio [HR], 1.00; 95% CI, 0.94–1.05; adjusted HR, 1.04; 95% CI, 0.98–1.10) (Table 6).

## Discussion

We investigated the prevalence of and risk factors for three prescribing cascades in an administrative data cohort of older adults with dementia. None of the three prescribing cascades investigated were common: bladder anticholinergics following cholinesterase inhibitors with 60 cases (0.2% of cohort), Parkinson’s disease medication following metoclopramide with 11 cases (0.04% of cohort), and any diuretic following CCB with 289 cases (1.0% of cohort) over the 5 years of study. Regression analysis demonstrated that risk of both bladder anticholinergics following cholinesterase inhibitors and diuretics following CCBs was associated with female sex. The risk of bladder anticholinergics following cholinesterase inhibitors was more common among those with a younger age at dementia diagnosis. In unadjusted regression analyses, diuretics following CCBs were more common in those whose dementia was diagnosed at an older age. Compared with people not taking the inciting medication, Cox regression suggested that bladder anticholinergics were less often used by those on cholinesterase inhibitors, suggesting that knowledge of this possible prescribing cascade is reducing anticholinergic pharmacologic management of urinary incontinence of those with dementia receiving cholinesterase inhibitors and did not identify CCB use as leading more frequently to diuretic prescription.

The cohort of NSSPBD studied approached 12% of the population of Nova Scotians over 65 years of age. This exceeds published estimates that 7–10% of older adults over age 65 live with dementia [52]. Nova Scotia has one of the oldest populations in Canada which may contribute to the higher prevalence of dementia in NSSPBD. There is also a sex imbalance in NSSPBD with an increased proportion of women (62.0% women), which is consistent with women being more likely to experience dementia than men [53]. The sex distribution varied by age, with more women than men with dementia at all age groups, skewing to greater proportions of women as age category increased which likely reflected women’s longer life expectancy.

An Ontario study by Gill et al. also investigated bladder anticholinergics prescribed to adults with dementia [9]. They found that older adults dispensed cholinesterase inhibitors were at a higher risk of being prescribed a bladder anticholinergic than those who were not, and found 916 prescribing cascade events over the 4 year period (HR 1.55; 95% CI (1.39 to 1.72)) [9]. The study authors did not comment on sex differences in use of bladder anticholinergics or in users of cholinesterase inhibitors. We had contrary findings in that bladder anticholinergic use was less common in users of cholinesterase inhibitors (HR 0.77; 95% CI (0.68–0.87)). This is promising that in the years since Gill’s study (which was run from 1999 to 2003) clinicians are more focused on avoiding this drug combination.

An Australian investigation into this same prescribing cascade found 36 cases over 4 years of study in a population of 4393 older adults with dementia (0.8% of their cohort compared to our finding of 0.2%). In the Australian study nearly half of the cases of the prescribing cascade had each medication prescribed by a different prescriber [54]. We did not capture data on prescriber so are unable to determine if different prescribers was a factor in our examination. This highlights the importance of encouraging a single prescriber, ideally each person’s primary care provider, to oversee treatment choices.

It is important that we avoid bladder anticholinergics regardless of cholinesterase inhibitor use in older adults with dementia. Bladder anticholinergics do inhibit the desired effect of cholinesterase inhibitor treatment [55] but also can lead to worsening cognitive and functional outcomes in older adults with dementia [56, 57]. Bladder anticholinergics are included on Beers’ list and the STOPP criteria [58, 59] as not appropriate for older adults with dementia. Additionally, women and those of younger age had an increased risk of bladder anticholinergic therapy following cholinesterase inhibitors. It is unclear from this analysis if this is because this is the population at greatest risk of the adverse effect of urinary incontinence from cholinesterase inhibitors, or those least likely to accept this adverse effect, or those most likely to request treatment for incontinence. This provides a starting point for interventions that could be used to address this prescribing cascade or identify the population that needs consideration for management of urinary incontinence. While bladder anticholinergics were not more common in those using cholinesterase inhibitors compared to those not using cholinesterase inhibitors, they are still being used against guideline recommendations. So, while this is not a large-scale problem it is of consideration for those caring for older adults with dementia.

The prescribing cascade of Parkinson’s Disease medication being initiated after metoclopramide was rare in our study population. A low use of metoclopramide was identified, only 1038 individuals (3.6%) and duration of use was short (mean of 73 days), so only a small portion of the cohort would have been at risk of developing movement-related adverse effects. The low incidence of this prescribing cascade in NSSPBD compares favourably with other jurisdictions. In a Korean population of adults over 60 years of age, those prescribed metoclopramide were about three times more likely to be prescribed levodopa than those who were not (OR 3.04; 95% CI (2.46–3.77) [4]. They also demonstrated that the odds of levodopa prescription increased with increasing duration of metoclopramide treatment (2.82 times for days 1–19 and 4.14 times for > 20 days; both odds ratios were adjusted for age, sex and exposure to antipsychotic medications) [4]. In a case-control study of New Jersey Medicaid program patients aged 65 years or older, those taking metoclopramide were also three times more likely to begin using a drug containing levodopa than patients not taking metoclopramide (OR 3.09; 95% CI (2.25 to 4.26)) [60]. It is reassuring that this prescribing cascade is not common in our cohort. It is also reassuring that metoclopramide was not commonly used and only for short durations. This may reflect use of metoclopramide for palliation or oncology purposes rather than chronic use for gastrointestinal syndromes.

In our cohort, 289 individuals (1.0%) met the criteria for the prescribing cascade where diuretics were used to treat CCB-induced pedal edema. This level of the prescribing cascade is consistent with population based estimates of 2–25% of CCB users experiencing the adverse drug effect of pedal edema [30] and Canadian studies of adults initiating CCB therapy demonstrating that between 4.6 and 9.5% initiate diuretic therapy shortly thereafter [5, 6, 42]. CCB-induced pedal edema may result in a prescribing cascade rather than drug discontinuation as the drug-induced pedal edema may be mistaken for a symptom of heart failure or peripheral vascular disease which are both common in older adults. Unfortunately, the primary mechanism of edema formation secondary to CCB use is arteriolar dilatation, not fluid overload, so a loop diuretic is not likely to resolve the problem [37]. More concerning is the potential complications from initiation of an unneeded loop diuretic including electrolyte monitoring, urinary incontinence, falls, and acute kidney injury. Diuresing euvolemic older adults experiencing CCB-induced edema places them at risk of dehydration, falls, urinary incontinence, acute kidney injury, electrolyte imbalance and many other consequences of overdiuresis [32, 61–63] which may be more serious for older adults with dementia who may be unable to manage their medications, hydration, or continence independently. The requirement of careful attention to hydration and possibly supplemental potassium may be far more challenging for older adults with dementia to manage than those with intact cognition and may increase caregiver burden for those reliant for support in medication management.

Women have been shown to be more likely to experience peripheral edema after treatment with CCB [30]. This was also identified in our population with women being at increased risk of the prescribing cascade in both adjusted and unadjusted analysis. In considering the reason women may be more sensitive to the adverse event of pedal edema, it may be due to the nature of feminine footwear which makes pedal edema easier for women to detect or more challenging for them to manage. Age was not statistically significantly associated with the CCB prescribing cascade in the multivariate regression suggesting that it was female sex that acted as a confounder in the significant association with age in the univariate analysis driven by the older age of the women in the cohort.

Research using administrative data has several inherent limitations. The identification of individuals with dementia presents its own challenges, though these were mitigated by leveraging previous work of the Nova Scotia Dementia Strategy [40]. Dementia likely remains underdiagnosed in Nova Scotia, so despite having access to a validated method of identifying those with dementia through administrative data we were unable to capture older women and men who have dementia but have not had a diagnosis recorded in the medical administrative record or did not participate in the Nova Scotia Seniors' Pharmacare program which limits generalizability of results. We did not capture any adults with dementia under the age of 65 as we were unable to examine drug use in these cases. The majority of older adults (approximately 63%) [39] in Nova Scotia subscribe to Seniors' Pharmacare, though Seniors' Pharmacare subscribers and non-subscribers may differ in important ways such as prior employment history (leading to use of private drug insurance rather than the public Pharmacare system) and hence socioeconomic status. We also were unable to identify which NSSPBD resided in a long-term care facility because this data was not captured in the administrative data until after the period of observation. We were however able to ascertain if they resided in an urban or rural location. We had limited ability to describe the clinical picture of the individual patients and we did not have data on medication indication, dose or directions. This includes not knowing if the patient had an indication that would make the second treatment appropriate. We assumed dispensing was equivalent to use which is not necessarily true. This is particularly meaningful for metoclopramide which can be used on an as needed basis. When examining the prescribing cascade, we did not have access to use of doctor-provided samples, medications purchased outside of the Pharmacare drug insurance program, or those purchased without a prescription. We also were unable to tell if the two drugs in the prescribing cascade had been prescribed by the same or different prescribers. We were only able to examine dates representing fill dates by the pharmacy and recognize this does not necessarily represent when the prescriptions were actually used by the patients. There were also limitations with respect to missing data, most importantly that a small percentage (0.8%) were missing data on sex. There are also possibly errors in the entered data as with any administrative dataset. Some of these issues may have been mitigated by attempting to collect data that included more clinical details via ICD 9/10 codes or details of hospital or emergency department admissions. Despite these limitations, the NSSPP data provided a unique and powerful opportunity to evaluate prescribing in a substantial portion of the older adults living with dementia in Nova Scotia.

## Conclusions

The prescribing cascade of bladder anticholinergics being used to treat cholinesterase inhibitor induced urinary incontinence occurred in 0.2% of our study population over a five-year period. Though rare overall, this prescribing cascade occurred more often for women, suggesting that sex-specific monitoring and management for cholinesterase inhibitor-induced urinary incontinence may be warranted. The incidence of the prescribing cascade of Parkinson’s Disease medications following metoclopramide-induced movement adverse events was rare. This is a positive finding for prescribing in Nova Scotia and suggests that prescribers are aware of and avoiding this prescribing cascade. Of NSSPBD on a CCB, 6% initiate a diuretic within 6 months of the CCB therapy. This is a common prescribing cascade (1.7%) and older adults with dementia would benefit from identification of this potential prescribing complication and CCB dose reduction or discontinuation by preventing unneeded diuretic use and potential complications such as falls, dehydration and urinary incontinence. This investigation only examined three of the many prescribing cascades that may affect older adults with dementia. Maintaining one prescriber is likely helpful in preventing occurrence of the prescribing cascade as there is consistent oversight for prescribing for each patient. Continued attention to prescribing cascades and their sex-specific incidence will be important to improve health care and outcomes for older adults with dementia.

## Data Availability

Data is held by Health Data Nova Scotia, an agent of the custodian (Department of Health and Wellness), and access requests can be handled by the corresponding author in consultation with the data custodian and agent.

## Abbreviations

ATC: Anatomical Therapeutic Chemical
CCB: Calcium Channel Blocker
DAD: Canadian Institute for Health Information – Discharge Abstract Database
HDNS: Health Data Nova Scotia
ICD: International Classification of Diseases Clinical Modification
MED: Physician’s Billings
MSI: Medical Services Insurance
NSSPBD: Nova Scotia Seniors' Pharmacare Beneficiary with dementia
PHARM: Seniors' Pharmacare
VITAL: Vital Statistics
